# Computational Elucidation of a Monobody Targeting the Phosphatase Domain of SHP2

**DOI:** 10.3390/biom15020217

**Published:** 2025-02-02

**Authors:** Yang Wang, Xin Qiao, Ruidi Zhu, Linxuan Zhou, Quan Zhang, Shaoyong Lu, Zongtao Chai

**Affiliations:** 1 Medicinal Chemistry and Bioinformatics Center, Shanghai Jiao Tong University School of Medicine, Shanghai 200025, China; spursanity@sjtu.edu.cn (Y.W.);; 2 Key Laboratory of Protection, Development and Utilization of Medicinal Resources in Liupanshan Area, Ministry of Education, Peptide & Protein Drug Research Center, School of Pharmacy, Ningxia Medical University, Yinchuan 750004, China; 3 Department of Hepatic Surgery, Shanghai Geriatric Medical Center, Shanghai 201104, China; 4 Department of Liver Surgery and Transplantation, Liver Cancer Institute and Zhongshan Hospital, Fudan University, Shanghai 200032, China

**Keywords:** Src homology 2 domain-containing phosphatase 2, Mb11, molecular dynamics simulation, targeted drug design, network analysis

## Abstract

Src homology 2 (SH2) domain-containing phosphatase 2 (SHP2) is a key regulator in cellular signaling pathways because its dysregulation has been implicated in various pathological conditions, including cancers and developmental disorders. Despite its importance, the molecular basis of SHP2’s regulatory mechanism remains poorly understood, hindering the development of effective targeted therapies. In this study, we utilized the high-specificity monobody Mb11 to investigate its interaction with the SHP2 phosphatase domain (PTP) using multiple replica molecular dynamics simulations. Our analyses elucidate the precise mechanisms through which Mb11 achieves selective recognition and stabilization of the SHP2-PTP domain, identifying key residues and interaction networks essential for its high binding specificity and regulatory dynamics. Furthermore, the study highlights the pivotal role of residue C459 in preserving the structural integrity and functional coherence of the complex, acting as a central node within the interaction network and underpinning its stability and efficiency. These findings have significantly advanced the understanding of the mechanisms underlying SHP2’s involvement in disease-related signaling and pathology while simultaneously paving the way for the rational design of targeted inhibitors, offering significant implications for therapeutic strategies in SHP2-associated diseases and contributing to the broader scope of precision medicine.

## 1. Introduction

**Figure 1:**
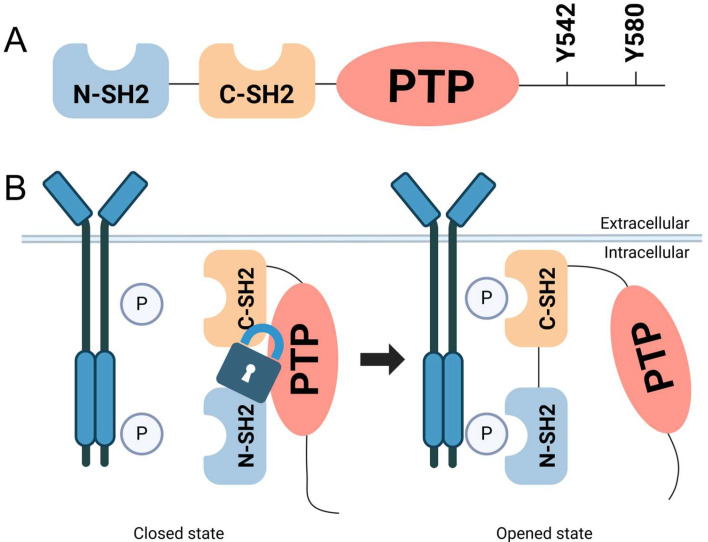
SHP2 protein structure and its activation mechanism. (**A**) SHP2 protein contains two SH2 domains, N-SH2 (blue) and C-SH2 (orange), followed by a central PTP domain (red). The C-terminal region contains phosphorylation sites, including Y542 and Y580, which play critical roles in SHP2’s regulatory functions. (**B**) The left part (closed state) depicts SHP2 in its autoinhibited conformation. In this state, the N-SH2 domain interacts with the PTP domain, blocking the active site and preventing catalytic activity. This interaction functions as a ‘lock’, maintaining SHP2 in a dormant state. The right part (opened state) illustrates SHP2 activation upon stimulation by external signals, such as growth factors or cytokines. Phosphotyrosine-containing ligands bind to the SH2 domains, inducing a conformational change that releases the N-SH2 domain from the PTP active site. This structural rearrangement exposes the catalytic center of the PTP domain, enabling enzymatic activity.

Src homology 2 (SH2) domain-containing phosphatase 2 (SHP2) is a non-receptor protein tyrosine phosphatase that plays an essential role in regulating signaling pathways involved in metabolism, proliferation, differentiation, migration, transcription, and oncogenesis [1,2,3]. Structurally, SHP2 comprises two tandem SH2 domains, a central phosphatase catalytic (PTP) domain, and a C-terminal region enriched with multiple functional phosphorylation sites [4] (Figure 1A). The N-terminal SH2 (N-SH2) and C-terminal SH2 (C-SH2) domains cooperate to mediate interactions with phosphotyrosine-containing ligands or adaptor proteins. Under basal conditions, the N-SH2 domain forms a non-covalent interaction with the PTP domain, maintaining SHP2 in an autoinhibited conformation [4,5]. Upon stimulation by external signals such as growth factors or cytokines, bidentate phosphotyrosine ligands bind to the SH2 domains, inducing a conformational change that releases the N-SH2 domain from the PTP active site. This structural rearrangement exposes the catalytic center of the PTP domain, enabling enzymatic activation [6,7] (Figure 1B).

The SHP2-PTP domain is critically involved in oncogenesis [8,9,10]. In breast and gastric cancers, SHP2 enhances RAS/ERK signaling by dephosphorylating inhibitory regulatory sites on adaptor proteins such as Gab1, thereby promoting cellular proliferation and resistance to targeted therapies [11,12,13,14]. In non-small cell lung cancer (NSCLC), PTP activity mediates resistance to EGFR inhibitors, such as osimertinib, by modulating the PI3K/AKT signaling pathway [15,16]. Additionally, activating mutations in SHP2, such as E76K, disrupt the autoinhibitory interaction between the N-SH2 and PTP domains in acute myeloid leukemia (AML), resulting in constitutive PTP activation and accelerated leukemic cell proliferation [9,17]. Moreover, the SHP2-PTP domain also plays a pivotal role in immune evasion [18,19]. Within the PD-1 signaling pathway, SHP2 suppresses T-cell activation by dephosphorylating critical components of the T-cell receptor (TCR) complex, including CD28 and ZAP70, thereby impairing the host’s anti-tumor immune response [20,21,22]. In solid tumors such as melanoma and hepatocellular carcinoma (HCC), SHP2 activity positively correlates with PD-L1 expression, further underscoring its central role in modulating the tumor immune microenvironment [23,24].

Beyond cancers, the SHP2-PTP domain is implicated in the pathogenesis of non-malignant diseases. Gain-of-function mutations, such as D61G and E76K in Noonan syndrome, lead to the constitutive activation of the RAS signaling pathway, disrupting tightly regulated developmental processes [25]. In contrast, loss-of-function mutations associated with LEOPARD syndrome impair SHP2 phosphatase activity, resulting in dysregulated signaling and developmental abnormalities [26].

Despite substantial progress in elucidating the role of the SHP2-PTP domain, the molecular mechanisms remain poorly understood. Recently, a monobody, Mb (SHP2PTP_11, hereafter referred to as Mb11), was identified as a highly specific binder of the SHP2-PTP domain [27], which may serve as a valuable tool for elucidating its regulatory mechanisms. Mb11, as demonstrated by Biolayer Interferometry (BLI), exhibits exceptional binding affinity for SHP2-PTP, with a dissociation constant (K_D_) of 2.7 nM. Notably, Mb11 displayed significantly reduced binding affinity to the SHP2-PTP(C459S) mutant, with a K_D_ value of 120 nM. This striking difference offers a critical opportunity to investigate the molecular determinants underlying Mb11’s interaction with SHP2-PTP.

Molecular dynamics (MD) simulations have become an indispensable tool for elucidating dynamic conformational changes and protein–protein interaction mechanisms at atomic resolution [28,29]. In this study, large-scale MD simulations were conducted to systematically explore the interaction mechanism between Mb11 and the PTP domain, with a specific focus on how Mb11 influences the conformation and dynamic properties of the PTP active site. The research aims to identify the key amino acid residues involved in Mb11-PTP interactions and to elucidate the primary forces stabilizing the Mb11-SHP2-PTP complex. These findings will provide critical molecular-level insights into Mb11’s mechanism of action and expand the theoretical framework for understanding SHP2 signaling regulation. Moreover, the study will provide a mechanistic basis for the rational design of potent SHP2 inhibitors and facilitate a deeper understanding of their regulatory pathways.

## 2. Results

### 2.1. Mb11 Targets the Deep Catalytic Pocket of the PTP Domain

**Figure 2:**
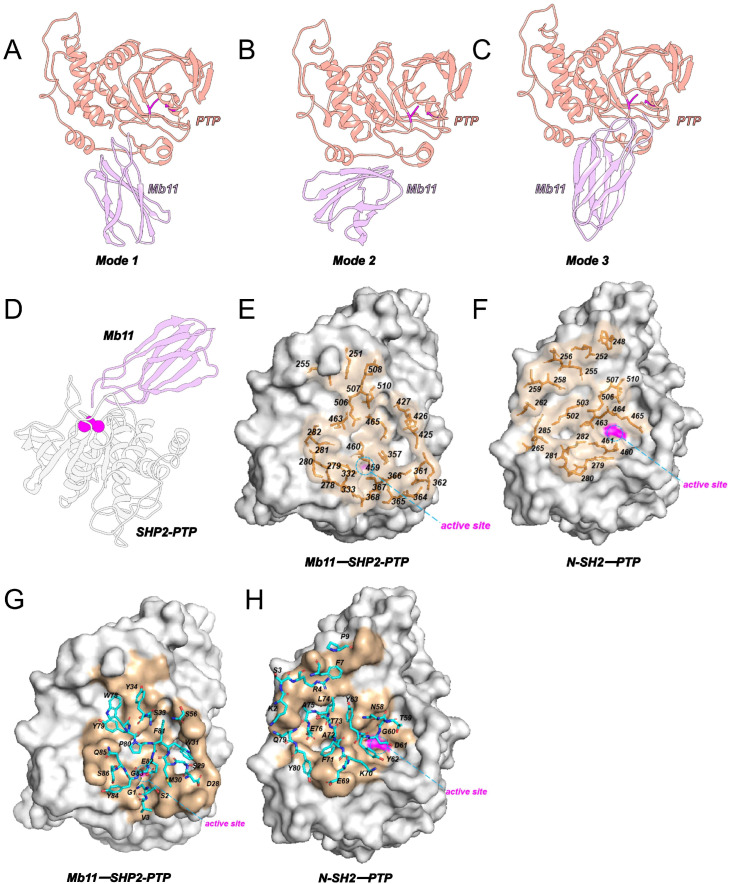
Static structural analysis of Mb11-SHP2-PTP. (**A**–**C**) Protein docking performed using the ClusPro2 server identified three potential binding modes of Mb11 with SHP2-PTP. Mb11 is shown in purple, SHP2-PTP in red, and the disulfide bond between C367 and C459 is highlighted in magenta. (**D**) The Mb11-SHP2-PTP complex is depicted, with Mb11 represented in purple and SHP2-PTP in white to enhance visual clarity. The critical residue, C459, is highlighted as a magenta sphere. (**E**) Detailed view of the Mb11-SHP2-PTP interaction interface, emphasizing critical residues within the defined epitope. PTP is depicted as a surface model, with the epitope region shown in wheat and rendered at 50% transparency. Residues identified as being within 4 Å of Mb11 heavy atoms are represented as orange sticks and labeled. The active site is outlined with a dashed circle, while the surface of C459 is highlighted in magenta. (**F**) Zoomed-in representation of the N-SH2-PTP interaction within the autoinhibited SHP2 structure, showcasing key PTP residues located within 4 Å of N-SH2 heavy atoms. Labeling conventions align with those used in (**E**). (**G**) Magnified view of the Mb11-SHP2-PTP interface, focusing on residues comprising the Mb11 paratope. PTP is shown as an opaque wheat-colored surface, with paratope residues (defined as those within 4 Å of PTP heavy atoms) displayed as sticks and labeled. The active site is marked with a dashed circle, and the C459 surface is highlighted in magenta. (**H**) Close-up depiction of the N-SH2-PTP interface within the autoinhibited SHP2 structure, concentrating on N-SH2 residues within 4 Å of PTP heavy atoms. Labeling methods follow those in (**G**). (**E**–**H**) are consistently oriented to facilitate comparative structural analysis.

Protein–protein docking analysis identified three potential binding modes for the Mb11-SHP2-PTP complex [30,31], labeled sequentially as Modes 1–3 (Figure 2A–C), which were subsequently used to construct models of the C459S mutant complex. Molecular Mechanics/Generalized Born Surface Area (MM/GBSA) calculations were performed to evaluate the Gibbs binding free energy ($\Delta G_{binding}$) of Modes 1–3 and their corresponding C459S mutants (Tables S1–S3) [32], ultimately identifying the most stable binding conformation (Figure 2C). The $\Delta G_{binding}$ of wild-type Mb11-SHP2-PTP was −48.43 ± 11.97 kcal/mol, whereas the $\Delta G_{binding}$ of C459S mutant was −37.11 ± 10.62 kcal/mol (Table S3). The mutation of the key catalytic site residue significantly weakens the binding affinity of Mb11.

Structural analysis of the final binding conformation revealed that Mb11 is directly positioned within the catalytic site of the PTP domain (Figure 2D), further supporting its role as a competitive inhibitor that blocks substrate access to the active site. Additionally, the binding epitope of Mb11 on SHP2-PTP shares certain similarities with the interface between the PTP and N-SH2 (Figure 2E,F) domains in the full-length SHP2 structure. However, Mb11’s binding is relatively more focused on the deep catalytic pocket of the PTP domain. Despite targeting similar surface regions, Mb11 and N-SH2 exhibit significant differences in the types of amino acids involved in binding (Figure 2G,H). Specifically, Mb11 does not mimic the PTP binding interface of N-SH2 but instead employs a distinct recognition pattern to interact with the catalytic surface. This unique binding mode may serve as the basis for Mb11’s specific interaction with SHP2-PTP, which merits further investigation.

### 2.2. Mb11 Significantly Enhances the Stability of SHP2-PTP

Large-scale MD simulations were employed to investigate the differential binding of Mb11 to wild-type and C459S mutant SHP2-PTP. To ensure clarity and consistency, the residue sequence and secondary structure of Mb11-SHP2-PTP are provided in Figure S1, which serve as the reference standard for subsequent discussions of residue-level interactions and structural analyses.

To assess the overall stability of the simulated systems, the root-mean-square deviations (RMSDs) of Cα atoms relative to the initial structure were calculated for each system (Figure S2A). RMSD analysis revealed that all systems gradually reached equilibrium after approximately 300 ns. Subsequent analyses focused on the trajectories within the equilibrium state. Further calculations showed that the RMSD value for the wild-type Mb11-SHP2-PTP complex was 3.53 ± 0.16 Å, whereas that of the C459S mutant was 5.69 ± 0.59 Å. This indicates that the wild-type complex exhibited lower dynamic fluctuation and a more stable binding mode, preliminarily supporting the high affinity of Mb11 for SHP2-PTP.

Next, the root-mean-square fluctuations (RMSFs) of Cα atoms relative to their initial positions were calculated for each residue (Figure S2B). The RMSF analysis revealed a generally similar distribution pattern across systems, with the wild-type Mb11-SHP2-PTP complex exhibiting consistently lower RMSF values. To further explore the relationship between residue-level flexibility and binding stability, residues involved in the Mb11-SHP2-PTP interface, as identified from the static structure (Figure 2E,G), were highlighted on the RMSF plot (blue). These interface residues in the wild-type complex displayed notably lower RMSF values, underscoring their role in stabilizing protein–ligand interactions.

**Figure 3:**
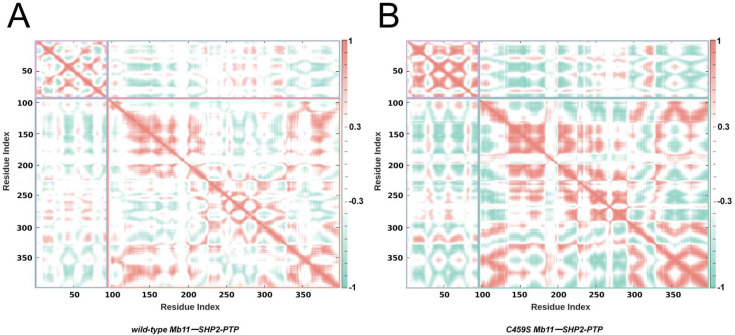
Comparative analysis of dynamic behavior in wild-type and mutant Mb11-SHP2-PTP complexes. (**A**) DCCMs for wild-type Mb11-SHP2-PTP. (**B**) DCCMs for C459S mutant complex. The purple box denotes intra-Mb11 correlated motions, the red and green boxes represent intra-SHP2-PTP correlations in the wild-type and mutant systems, respectively, and the blue box indicates inter-molecular correlations between Mb11 and SHP2-PTP. The color scale is displayed on the right. Interactions with absolute correlation coefficients below 0.3 are shown in white to improve clarity.

To further elucidate the distinct conformational dynamics triggered by the C459S mutation, dynamic cross-correlation matrices (DCCMs) were calculated for the Cα atoms (Figure 3A,B). In the DCCM plots, the diagonal regions represent the motion of residues relative to themselves, while off-diagonal regions indicate the correlated motions between different residues. Based on the color-coding scheme, red signifies positively correlated motions between residues, while green denotes anti-correlated motions. To facilitate interpretation, key regions were highlighted using colored boxes in the matrix plots. The purple box represents the internal correlated motions within Mb11, the red and green boxes correspond to the intra-molecular correlated motions within wild-type and C459S mutant SHP2-PTP, respectively, and the blue box indicates inter-molecular correlated motions between Mb11 and SHP2-PTP. The results revealed that both intra- and inter-molecular correlated motions in the wild-type Mb11-SHP2-PTP complex were significantly weaker than those in the C459S mutant. This diminished correlation suggests that the wild-type complex exhibits more coordinated and stable overall dynamics, further supporting its superior binding dynamics.

### 2.3. Wild-Type Mb11-SHP2-PTP Exhibits a More Compact Conformation

**Figure 4:**
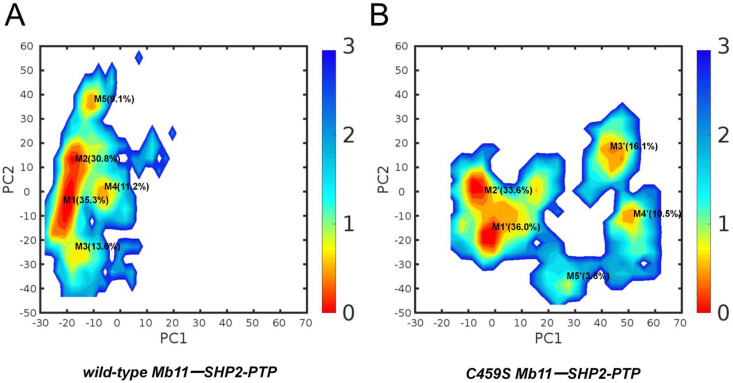
PCA and free energy landscapes of wild-type and C459S mutant Mb11-SHP2-PTP complexes. (**A**,**B**) depict the free energy landscapes of the wild-type and C459S mutant systems, respectively, based on PC1 and PC2. Free energy values are expressed in kcal/mol, with the corresponding color scale shown on the right. K-means clustering of the PCA projections identifies the five most dominant conformational clusters for each system, with their respective occupancy percentages annotated near the cluster centroids.

To understand the conformations during the simulations, principal component analysis (PCA) was performed on the wild-type and C459S mutant Mb11-SHP2-PTP complexes (Figure 4A,B). The two systems were projected onto a shared PCA space to ensure direct comparability of their conformational landscapes. By extracting the two most significant principal components (PC1 and PC2), the high-dimensional conformational space was reduced to a two-dimensional (2D) distribution map for each system. The results indicate that the wild-type Mb11-SHP2-PTP complex exhibits significant conformational convergence, with its distribution concentrated within a narrow range along PC1, forming compact cluster structures. This distribution pattern suggests that the wild-type complex has lower conformational fluctuations in the conformational space, tending towards a more stable conformation and a tighter energy state. In contrast, the conformational distribution of the C459S mutant complex shows a marked expansion in coverage along PC1, displaying a highly dispersed distribution pattern. Notably, M3’ and M4’ deviate significantly from the primary clusters and are located at the distal regions of the PC1 and PC2 space, highlighting the heterogeneity of the conformational space. This dispersed distribution pattern clearly indicates that the C459S mutant complex occupies a higher free-energy state within the conformational space.

Next, we investigated the PCA eigenvalue distributions of the two systems to further elucidate their differences in conformational dynamic properties (Figure S3A,B). The cumulative variances of PC1 and PC2 for the wild-type complex are relatively low (40.1% and 27.1%), with an eigenvalue decay curve displaying a gradual slope, indicative of more evenly distributed conformational motions across multiple PCs. This distribution reflects the greater conformational stability and the restricted conformational space of the wild-type complex. In contrast, the C459S mutant exhibits significantly higher variances for PC1 and PC2 (73.0% and 13.5%), with a steep eigenvalue decay after PC1. This sharp decline indicates that the mutant’s motions are predominantly concentrated in a single dominant principal direction, suggesting larger amplitude motions, enhanced conformational flexibility, and broader sampling of the conformational space. These findings further substantiate that the mutant explores a more extensive conformational landscape, while the wild-type remains confined to a narrower free energy basin, indicative of superior stability.

Figure S4A,B illustrates the five representative conformations of each system complemented by RMSD values obtained from structural alignments (Figure S4C,D), which, from a new perspective, validate the differences in conformational flexibility and stability between both systems. Specifically, the C459S mutant displays markedly higher RMSD values and greater structural divergence among its representative conformations. In contrast, the wild-type Mb11-SHP2-PTP complex exhibits lower RMSD values and more similar representative conformations, indicating a more compact and stable conformational state. These findings reinforce the distinct advantage of the wild-type complex in terms of conformational stability.

To further investigate the conformational properties of wild-type and C459S mutant Mb11-SHP2-PTP complexes, the Defined Secondary Structure of Proteins (DSSP) method was used to analyze dynamic secondary structure changes throughout the simulation (Figure S5A,B). Comparative results indicated that in the wild-type Mb11-SHP2-PTP system, α-helices and β-sheets exhibited a higher proportion and maintained stronger temporal continuity. This suggested a more compact and stable overall conformation. In contrast, the C459S mutant showed an increase in bend, turn, and disordered regions (denoted as ‘none’ in Figure S5). These unstable structures were more widely distributed throughout the mutant system and persisted for extended periods, reflecting looser and more dynamic conformational characteristics. These trends corroborated the PCA findings, further confirming the distinct conformational stability and dynamics of both systems.

### 2.4. Robust Inter-Protein Interactions Drive an Observed ‘Approaching’ Trend

To quantitatively evaluate the similarity of dynamic behavior between the two complexes, we performed a Root Mean Square Inner Product (RMSIP) analysis on the first 10 PCs of the two systems. The analysis revealed an RMSIP value of 0.33, which is significantly lower than the ideal value of 1.0, indicating substantial differences in the overall dynamic behavior within the principal subspaces of the two systems. Further analysis of the overlap matrix showed that the overall degree of overlap between the PCs of the two systems was generally low. As illustrated in Figure S6, most overlap values are close to 0, indicating that the two systems exhibit markedly different dynamic behavior along their major motion directions. The limited similarity between the PCs suggests that the mutation has profoundly impacted the global dynamic modes of the system, significantly altering the characteristics of its dynamical subspace.

**Figure 5:**
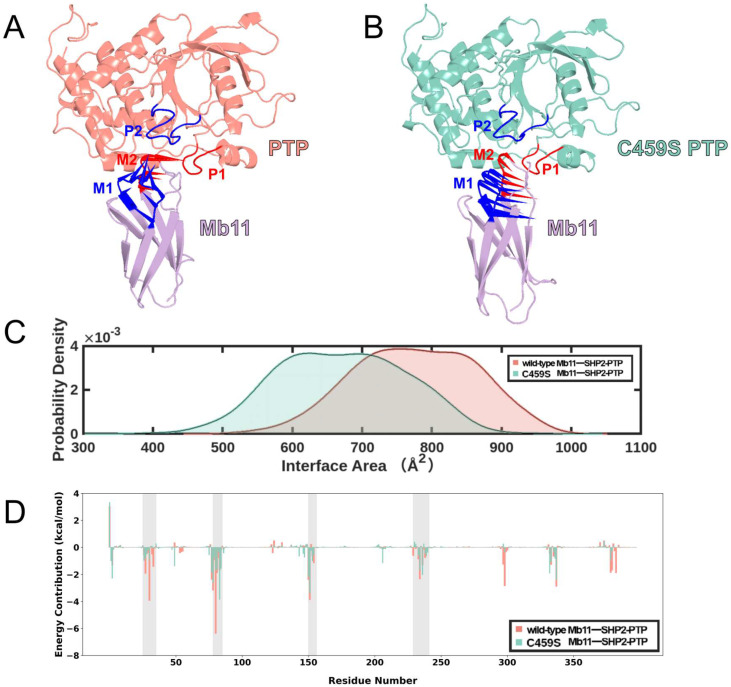
Inter-protein interactions drive the ‘approaching’ trend in Mb11-SHP2-PTP systems. (**A**,**B**) Porcupine plots illustrate the primary motion along PC1 in the wild-type and C459S mutant Mb11-SHP2-PTP systems. Mb11 is shown in purple, the wild-type SHP2-PTP in red, and the C459S mutant SHP2-PTP in green. Loops M1 and P2 are marked in deep blue, and Loops M2 and P1 are marked in vivid red, highlighting the distinct motion patterns between the two systems. (**C**) Probability distribution of the interface area in the wild-type (red) and C459S mutant (green) systems during simulations. (**D**) Per-residue decomposition of binding free energy in the wild-type (red) and C459S mutant (green) systems. Key residue regions, including Loops M1, M2, P1, and P2, are highlighted with a semi-transparent gray background.

To graphically illustrate the dominant motions within different regions of Mb11-SHP2-PTP, PC1 was mapped onto the initial structure for each system (Figure 5A,B). The analysis revealed an ‘approaching’ motion at the interface of wild-type Mb11-SHP2-PTP, particularly between loop M1 and P2, as well as loop M2 and P1 (specific residue composition shown in Table S4), indicating enhanced interaction and structural convergence. In contrast, the C459S mutant system exhibited a weak ‘departing’ motion, characterized by reduced inter-regional interactions and an increased spatial separation between these loops.

Building on the observed ‘approaching’ dynamics, we hypothesized that the protein–protein interface (PPI) in the wild-type Mb11-SHP2-PTP complex would be larger. As illustrated in Figure 5C, the wild-type complex displayed a prominent peak at an interface area of approximately 750 Å^2^, indicating a more uniform and tightly packed PPI. In contrast, the C459S mutant exhibited a bimodal distribution, with two dominant peaks around 625 Å^2^ and 700 Å^2^, reflecting a less homogeneous and potentially less stable interface.

Generally, larger interface areas are linked with stronger binding interactions due to lower protein–protein interaction energies. To further quantify this relationship, we employed the Molecular Mechanics Poisson–Boltzmann Surface Area (MM/PBSA) method to calculate the $\Delta G_{binding}$ between Mb11 and SHP2-PTP (Table 1) [32]. The results showed that the wild-type complex exhibited a more favorable $\Delta G_{binding}$ of −57.79 ± 13.04 kcal/mol, compared to −49.09 ± 11.70 kcal/mol for the C459S mutant. The stronger binding free energy in the wild-type complex is consistent with its larger interface area and highlights the critical role of stable protein–protein interactions in maintaining structural integrity and functional specificity.

Next, we decomposed $\Delta G_{binding}$ on a per-residue mode (Figure 5D). In the wild-type Mb11-SHP2-PTP system, the intermolecular interaction network was strengthened either by enhancing favorable negative Gibbs free energy contributions or mitigating unfavorable positive contributions. Key interaction regions, including Loops M1, M2, P1, and P2, are highlighted (gray). Residues within these regions exhibited significantly negative energy contributions in the wild-type system, underscoring their pivotal role in supporting enhanced conformational stability and dynamic behavior.

### 2.5. Deciphering the Critical Interacting Residues That Drive the Observed Differences

**Figure 6:**
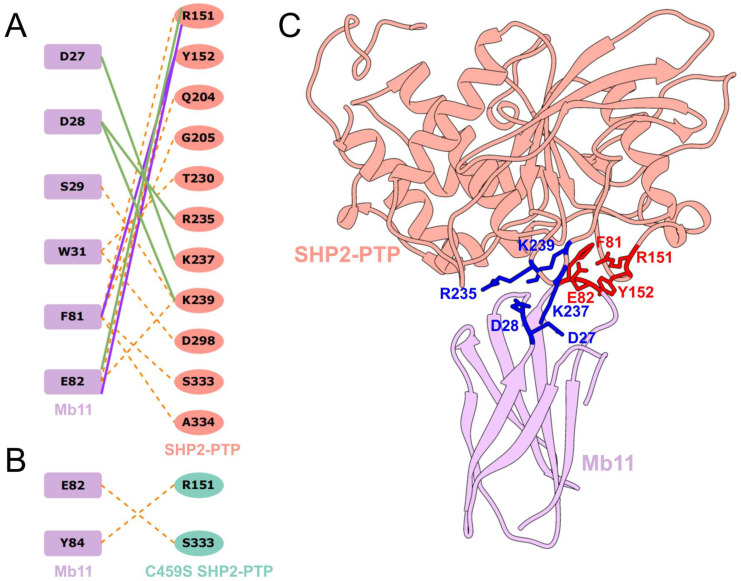
Key residue interactions between Mb11 and SHP2-PTP in wild-type and C459S mutant systems. Schematic representation of residue–residue interactions in the wild-type (**A**) and C459S mutant (**B**) Mb11-SHP2-PTP system. Purple blocks represent Mb11 residues, while red ovals and green ovals represent wild-type and C459S mutant SHP2-PTP residues, respectively. Dashed orange lines represent hydrophobic contacts, solid green lines represent salt bridges, and solid purple lines represent hydrogen bonds. (**C**) Three-dimensional structure showing the interaction interface between Mb11 (purple) and SHP2-PTP (red). Five key stable polar interactions are highlighted, with deep blue and vivid red residues illustrating the approaching trend between M1 and P2, and M2 and P1, respectively.

To preliminarily explore the molecular interaction mechanisms underlying the ‘approaching’ dynamics, we defined a ‘stable contact’ as a residue–residue interaction persisting for at least 70% of the total simulation time. The analysis revealed that in the wild-type Mb11-SHP2-PTP system, extensive stable hydrophobic interactions serve as the cornerstone of the overall interaction network (Figure 6A,B). Moreover, five key stable polar interactions were identified during the simulations (D27-K237, D28-R235, D28-K239, F81-Y152, and E82-R151). These residue pairs demonstrated not only close spatial proximity but also complementary electrostatic properties (Figure 6C), which explains the observed ‘approaching’ trend.

To comprehensively evaluate the influence of key interacting residues on the stability of the Mb11-SHP2-PTP complex, we performed a systematic analysis of salt bridges and hydrogen bonds across both systems (Table 2). The findings revealed that a small subset of highly persistent salt bridges and hydrogen bonds, with occupancies exceeding 50% in the wild-type system, constituted the core of the interfacial interaction network, whereas these interactions were markedly attenuated in the C459S mutant system.

In the wild-type complex, the salt bridge E82-R151 exhibited exceptional stability, with an occupancy of 77.8%, underscoring its pivotal role as a structural anchor driven by strong electrostatic attraction. Similarly, the salt bridge D27-K237 maintained an occupancy of 54.0%, while the hydrogen bond F81-Y152 demonstrated an even higher occupancy of 63.2%, collectively reinforcing the electrostatic framework. In stark contrast, these core interactions were significantly disrupted in the C459S mutant system. The occupancy of the salt bridge E82-R151 plummeted to 3.4%, and the hydrogen bond F81-Y152 was reduced to a mere 2.57%, highlighting the mutation’s profound destabilizing effect on the primary interaction network. Beyond these core interactions, secondary contributions from the salt bridges D28-R235 and D28-K239, as well as the hydrogen bond E82-R151 in the wild-type system, provided additional stabilizing support. Thus, we have elucidated the molecular mechanisms underpinning the stability of the wild-type Mb11-SHP2-PTP complex.

### 2.6. The Interaction Network Unveils the Driving Forces Behind the Binding Specificity

**Figure 7:**
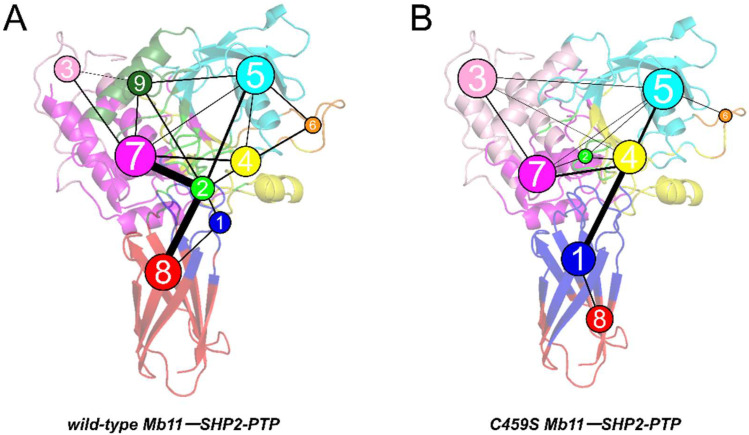
Community networks overlaid on the 2D structure of the wild-type (**A**) and C459S mutant (**B**) Mb11-SHP2-PTP systems. Each circle denotes a distinct community, with its size reflecting the number of residues it contains. The thickness of the lines connecting the circles represents the strength of intercommunity interactions.

Variation in polar interaction occupancy contributes to Mb11’s binding selectivity; however, the fundamental mechanisms driving this difference remain elusive. Therefore, Community Network Analysis (CNA) was performed using the Girvan–Newman algorithm [33]. Variational coupling between communities was quantitatively assessed across all simulation trajectories. Residues within 4.5 Å of each other for at least 75% of the simulation time were grouped into the same community, which were considered cooperative functional units within the overall protein structure. The community composition and their interconnections are visualized in Figure 7A,B.

Preliminary analysis reveals that Communities 1 and 8 primarily comprise Mb11 residues, while Communities 3–7 and 9 are largely composed of SHP2-PTP residues. However, in the wild-type complex, Community 2 occupies the protein interface and predominantly includes residues involved in key stabilizing interactions, serving as a central hub for interfacial connectivity. Community 2 forms strong connections with multiple other communities, particularly Communities 7 and 8, maintaining the integration and functional coordination of the interfacial network. In the mutant system, the composition and position of Community 2 change significantly. Community 2 shifts deeper into the SHP2-PTP structure, becoming entirely composed of SHP2-PTP residues, with most of its components reassigned to Communities 1 and 4. Moreover, the C459S mutation disrupts the hub-like functionality of Community 2, markedly weakening or even eliminating its connections with other communities. These changes underscore the critical differences in community organization between the systems, where the highly coordinated network in the wild-type provides a robust foundation for interfacial stability and binding efficiency.

Further analysis reveals that the C459S mutation induces significant changes in binding patterns. In the wild-type complex, C459 is located within Community 2, acting as a pivotal node in the electrostatic interaction and hydrogen bond network at the interface. This positioning reinforces the hub functionality of Community 2, allowing it to coordinate interactions across multiple communities, thereby enhancing interfacial coordination and network integrity. This network contributes to the superior dynamic stability and binding efficiency of the wild-type complex. In the mutant system, however, C459 is reassigned to Community 4, resulting in the loss of Community 2’s functionality. Post-mutation, the coordination of the interfacial electrostatic and hydrogen bond network is significantly weakened, with intercommunity connections relying solely on limited interactions between Communities 1 and 4. This leads to reduced residue integration at the interface and fragmentation of the protein–protein interaction network. These changes highlight the destabilizing impact of the C459S mutation on interfacial network stability and complex functionality.

In summary, C459 serves as a core hub within the interfacial electrostatic and hydrogen bond network, integrating key residues and coordinating interactions between communities to maintain the structural stability and dynamic coordination of the complex. The C459S mutation disrupts this critical hub, resulting in a significant reduction in interfacial network coordination and structural integrity and ultimately impairing the binding efficiency and functional specificity of the complex.

## 3. Discussion

SHP2 plays an indispensable role in cellular signal regulation, with aberrant activity of its PTP domain closely associated with various cancers and developmental disorders. However, the molecular mechanisms remain largely unexplored. This knowledge gap not only limits our understanding of the biological functions of SHP2 but also significantly hinders the development of efficient targeted therapeutics. To address this issue, this study employed the high-specificity monobody Mb11 in complex with the SHP2-PTP domain and its C459S mutant as the research model. Using large-scale MD simulations, we systematically investigated the binding mechanisms and dynamic behavior of Mb11 with SHP2-PTP.

Preliminary analyses revealed that the Mb11-SHP2-PTP complex exhibited greater overall conformational stability and reduced molecular flexibility compared to the C459S mutant. DCCM analysis demonstrated a tighter protein–protein interface interaction pattern in the wild-type complex. PCA analysis further supported this conclusion, showing a more concentrated low-energy state distribution and a dynamic ‘approaching’ trend at the interface of the wild-type complex. MM/PBSA calculations indicated that the more stable interface interactions in the Mb11-SHP2-PTP complex were the driving factors behind this ‘approaching’ trend. Key residues responsible for the robust interface interactions were determined through a systematic analysis of salt bridges and hydrogen bonds. Finally, the visualized community network highlighted the changes in network topology before and after mutation, further revealing the critical role of the C459 residue as a central hub in the interface network.

This study addressed the key scientific question of Mb11’s selective targeting of SHP2-PTP and systematically elucidated the molecular mechanisms underlying its binding specificity. Mb11 achieves high selectivity by precisely targeting the deep catalytic pocket of SHP2-PTP, a process dependent on multiple highly stable and functionally cooperative polar interactions at the interface, including the salt bridges D27-K237 and E82-R151, as well as the hydrogen bond F81-Y152. These interactions not only enhance intermolecular electrostatic attraction but also stabilize interface binding through the integration of a hydrophobic network. More importantly, C459 acts as a core hub in the interface network, playing an irreplaceable role in coordinating dynamic interactions between interface residues, maintaining network topology, and ensuring interfacial cooperativity. The integrity of its structure directly determines the high binding affinity and functional specificity of the complex, while its mutation disrupts these interaction networks, leading to a significant loss of interface coordination and binding efficiency. This study not only uncovers the molecular basis of Mb11-SHP2-PTP binding but also highlights the decisive role of key residues in interface functionality, providing valuable theoretical guidance for the rational design of SHP2-targeted therapeutics.

Future research should focus on optimizing the structural properties of the monobody to enhance its specificity for SHP2-PTP, particularly in pathological conditions associated with SHP2 dysfunction. Additionally, it is necessary to investigate the dynamic behavior and functional mechanisms of Mb11 in diverse physiological and pathological contexts to uncover its potential roles in regulating SHP2 signaling networks. This study provides critical molecular insights for the development of efficient SHP2-targeted inhibitors and deepens the understanding of SHP2’s pivotal role in disease-related signaling and pathological mechanisms, thereby laying a solid foundation for precision therapeutic strategies in cancer and other SHP2-associated diseases.

## 4. Materials and Methods

### 4.1. Simulation System Preparation and Conformational Screening

In this study, we constructed two simulation systems—the wild-type and C459S mutant Mb11-SHP2-PTP complexes—and determined the conformations through a multi-step screening and evaluation process.

First, the initial crystal structure of the Mb13-SHP2-PTP complex (PDB ID: 7TVJ) was obtained from the Protein Data Bank (PDB) [27]. Subsequently, Modeller 10.4 in USCF Chimera was used to add missing non-terminal residues and remove non-protein co-crystallized molecules [34], yielding the complete structures of Mb13, Mb11, and SHP2-PTP. To capture representative conformations, three sets of 1 μs MD simulations were conducted for the unbound structures of Mb11 and SHP2-PTP individually. Protein–protein docking was then performed using ClusPro 2.0 to generate reasonable docking models [30,31], followed by site-directed mutagenesis in UCSF Chimera to construct the corresponding C459S mutants. Finally, three sets of 1 μs MD simulations were conducted for both the wild-type and C459S mutant Mb11-SHP2-PTP systems, and energy evaluation and refinement were carried out using the MM/GBSA method.

### 4.2. MD Simulations

To investigate the protein dynamics under realistic environmental conditions, MD simulations were performed for both systems using AMBER 20. The initial parameters for energy minimizations and simulations were prepared using the Amber ff14SB force field via the Leap module [35,36,37,38]. Both systems were solvated in a truncated octahedral water box filled with transferable intermolecular potential three-point (TIP3P) water molecules, extending 12 Å from the protein surface [39,40,41]. Counterions were added to neutralize the overall charge of each system, ensuring an electrically neutral simulation environment. All MD simulations utilized the GPU-accelerated PMEMD module for computational efficiency [42]. Energy minimization was conducted in two stages to remove steric clashes and optimize the system. The first stage involved 50,000 steps of minimization, allowing non-protein components to move freely while restraining the protein atoms. In the second stage, all atomic movements were unrestricted, with an additional 100,000 steps performed using a combination of the steepest descent and conjugate gradient algorithms. Following minimization, the systems were gradually heated from 0 K to 310 K over 300 ps using Langevin dynamics in a canonical ensemble (NVT). Equilibration was then performed for 700 ps under the same conditions to stabilize the systems. Subsequently, production runs consisting of three independent 1 μs simulations for each system were carried out in an isothermal-isobaric ensemble (NPT) at 310 K and 1 atm pressure. The simulations employed a time step of 2 fs. Long-range electrostatic interactions were computed using the Particle Mesh Ewald (PME) method with a 10 Å cutoff [43,44]. To maintain system stability, all covalent bonds involving hydrogen atoms were constrained using the SHAKE algorithm [45,46].

### 4.3. Dynamic Cross-Correlation Matrix Analysis

DCCM analysis was conducted for both systems using the CPPTRAJ module in AMBER20 to evaluate residue–residue correlations. The normalized cross-correlation matrix was computed, with its elements representing cross-correlation coefficients [47], and derived using the following equation:

(1) $$C\left(i,j\right)=\frac{c\left(i,j\right)}{\sqrt{c\left(i,i\right)\times c\left(j,j\right)}}$$

In this equation, $C\left(i,j\right)$ denotes the cross-correlation coefficient between the fluctuations in the coordinates of Cα atoms *i* and j, ranging from −1 to 1. $c\left(i,j\right)$ represents the covariance of the coordinate fluctuations between Cα atoms *i* and j. $c\left(i,i\right)$ and $c\left(j,j\right)$ correspond to the variances of the coordinate fluctuations of Cα atoms *i* and j, respectively.

### 4.4. Principal Component Analysis and Free Energy Landscapes

PCA is a widely used statistical method in simulation studies to identify dominant protein dynamics and explain relevant phenomena. By simplifying a large set of interdependent variables (atomic coordinates) into a smaller set of independent variables, PCA facilitates the interpretation of complex motion patterns [48]. This is achieved by solving the eigenvalues and eigenvectors of the covariance matrix, thereby decomposing the observed motions into uncorrelated principal components (PCs). Each PC is represented as a linear combination of correlated variables, expressed by the following equation:

(2) $${PC}_{i}=\alpha_{i1}x_{1}+\alpha_{i2}x_{2}+\cdots +\alpha_{im}x_{m}=\sum_{j=1}^{m}\alpha_{ij}x_{j}$$

Among these eigenvectors, PC1 and PC2 capture the largest proportion of variance, reflecting the dominant motions in the MD simulations [49]. To enable meaningful comparisons across simulations, root mean square (RMS) fitting was performed using a common reference structure, effectively removing translational and rotational motions of the proteins.

The free energy landscape (FEL) serves as a powerful approach for investigating protein conformational ensembles and is derived using the following equation [50,51,52]:

(3) $$G_{i}=-k_{B}T\ln\frac{N_{i}}{N_{m}}$$

Here, $k_{B}$ represents the Boltzmann constant, and T is the temperature of the simulation system. $N_{i}$ and $N_{m}$ denote the populations of the *i*-th bin and the most populated bin, respectively. To generate the FELs, PC1 and PC2, RMSD values, and specific distances were utilized as reaction coordinates, with energy levels visualized through color-coded gradients.

### 4.5. Binding Free Energy Calculations

Binding free energies were calculated using the MM/PBSA method implemented in the MMPBSA.py plugin of AMBER20 [53]. This approach quantifies the Gibbs free energy changes upon ligand binding in binary and ternary systems. The total $\Delta G_{binding}$ was derived as follows:

(4) $$\Delta G_{binding}=\Delta G_{complex}-\Delta G_{ligand}-\Delta G_{receptor}$$

According to thermodynamic principles, the Gibbs free energy change in a solvated system consists of enthalpic (ΔH) and entropic (−TΔS) contributions. The enthalpic term is further decomposed into molecular mechanical energy ($\Delta E_{MM}$), representing interaction energies between the receptor and ligand, and the solvation free energy ($\Delta G_{sol}$), leading to the following expression:

(5) $$\;\Delta G_{binding}=\Delta E_{MM}+\Delta G_{sol}-T\Delta S$$

The molecular mechanical energy ($\Delta E_{MM}$) comprises three components: van der Waals ($\Delta E_{vdW}$), electrostatic ($\Delta E_{ele}$), and intramolecular energies ($\Delta E_{int}$) derived from bond, angle, and torsional terms, as follows:

(6) $$\;\Delta E_{MM}=\Delta E_{vdW}+\Delta E_{ele}+\Delta E_{int}$$

For the solvation term ($\Delta G_{sol}$), a Poisson–Boltzmann continuum solvent model was applied. This term is divided into polar ($\Delta E_{PB}$) and nonpolar ($\Delta E_{nonpolar}$) contributions, as follows:

(7) $$\;\;\Delta G_{sol}=\Delta E_{PB}+\Delta E_{nonpolar}$$

The nonpolar component ($\Delta E_{nonpolar}$) was calculated using a simplified linear relation with the solvent-accessible surface area (SASA), where γ was set to 0.00542 ${kcal\cdot mol}^{-1}\cdot {Å}^{-2}$ and the offset b was assigned a value of 0.92 ${kcal\cdot mol}^{-1},\;as\;follows$:

(8) $$\Delta E_{nonpolar}=\gamma \;*\;SASA+b$$

Notably, the entropy term (−TΔS) was excluded from the calculations due to the low conformational fluctuations observed in the systems, as indicated by their low RMSD values. Moreover, estimating the entropy using normal mode analysis or quasi-harmonic approaches would require significantly higher computational resources while introducing potential errors in systems with small conformational variations. The analysis thus focused on the relative free energy contributions of enthalpic and solvation terms, which are sufficient for comparing the binding free energies between similar systems.

### 4.6. Community Network Analysis

Using the NetworkView plugin in VMD, the correlation coefficient matrix $C\left(i,j\right)$ was computed to investigate intercommunity interactions and analyze the community network structure [54]. In this analysis, Cα atoms of both structures were treated as nodes, each corresponding to a residue. Edges were established between nodes if their pairwise distance remained within 4.5 Å for a minimum of 75% of the simulation duration. The edge weight was calculated using the following formula:

(9) $$d_{\left(i,j\right)}=-log\left(\left|C_{\left(i,j\right)}\right|\right)$$

where i and j denote the respective nodes. The Girvan–Newman divisive algorithm, applied using the gncommunities program, was utilized to segment the network into distinct communities [55]. Intercommunity interactions were evaluated based on betweenness centrality, which quantifies the role of a node as a connector or bridge between separate communities. To enhance the robustness of the analysis, communities comprising fewer than three residues were omitted from the final network visualization.

## Figures and Tables

**Table 1:** Binding free energies (kcal/mol) of wild-type and C459S mutant Mb11-SHP2-PTP systems computed by the MM-PBSA method.

Energy Component (kcal/mol)	Wild-Type Mb11-SHP2-PTP	C459S Mb11-SHP2-PTP
$\Delta G_{gas}$	−482.92 ± 67.09	−498.85 ± 95.77
$\Delta G_{solv}$	425.13 ± 59.68	449.76 ± 88.69
$\Delta G_{binding}$	−57.79 ± 13.04	−49.09 ± 11.70

**Table 2:** Systematic analysis of salt bridges and hydrogen bonds in both systems.

Residue Pair	Interaction Type	Wild-Type Mb11-SHP2-PTP	C459S Mutant Mb11-SHP2-PTP
F81-Y152	Hydrogen bond	63.20%	2.57%
E82-R151	Hydrogen bond	34.00%	1.13%
D27-K237	Salt bridge	54.00%	24.33%
D28-R235	Salt bridge	46.10%	19.80%
D28-K239	Salt bridge	36.00%	9.33%
E82-R151	Salt bridge	77.80%	3.40%

## Data Availability

Data are contained within the article and Supplementary Materials.

## Supplementary Materials

The following supporting information can be downloaded at: https://www.mdpi.com/article/10.3390/biom15020217/s1, Figure S1: Detailed residue sequence and secondary structure information of Mb11-SHP2-PTP complex; Figure S2: RMSD and RMSF analyses of wild-type and C459S mutant Mb11-SHP2-PTP complexes; Figure S3: PCA eigenvalue distributions for wild-type and C459S mutant Mb11-SHP2-PTP systems; Figure S4: Representative conformations of wild-type and C459S mutant Mb11-SHP2-PTP complexes, along with RMSD heatmaps illustrating the structural variations of the representative conformations; Figure S5: DSSP analysis of residue-specific secondary structure transitions in wild-type and C459S mutant Mb11-SHP2-PTP systems; Figure S6: Heatmap of the Overlap Matrix Between the First 10 PCs of wild-type and C459S mutant complexes; Table S1: Binding free energies (kcal/mol) of Mode 1 and its corresponding C459S mutant, calculated using the MM-GBSA method; Table S2: Binding free energies (kcal/mol) of Mode 2 and its corresponding C459S mutant, calculated using the MM-GBSA method; Table S3: Binding free energies (kcal/mol) of Mode 3 and its corresponding C459S mutant, calculated using the MM-GBSA method; Table S4: Detailed residue composition of Loops M1, M2, P1, and P2.
