# Supplementary material for: Computational Elucidation of a Monobody Targeting the Phosphatase Domain of SHP2
Source: Biomolecules. 2025 Feb 2;15(2):217. doi: 10.3390/biom15020217 (PMC11853358; doi:10.3390/biom15020217)
Supplement: Supplementary file 1 [file biomolecules-15-00217-s001.zip › biomolecules-3399099-supplementary.pdf]

Supplementary Information

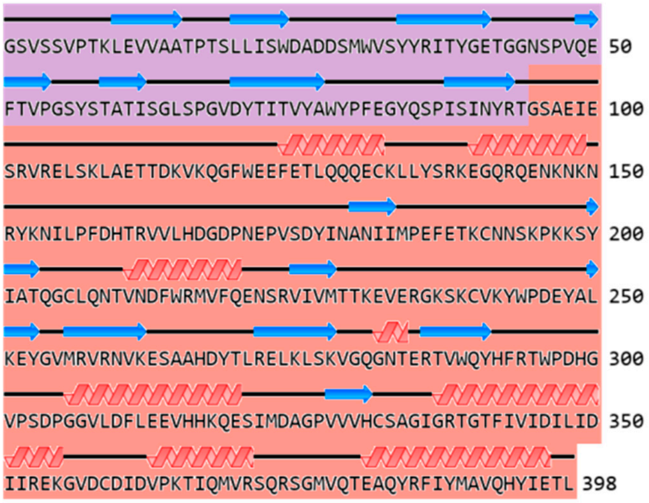

Figure S1. Detailed residue sequence and secondary structure information of Mb11–SHP2-PTP. α-helices are represented by red spirals, while β-sheets are shown as blue arrows. Mb11 is indicated with a purple background, while SHP2-PTP is highlighted with a red background.

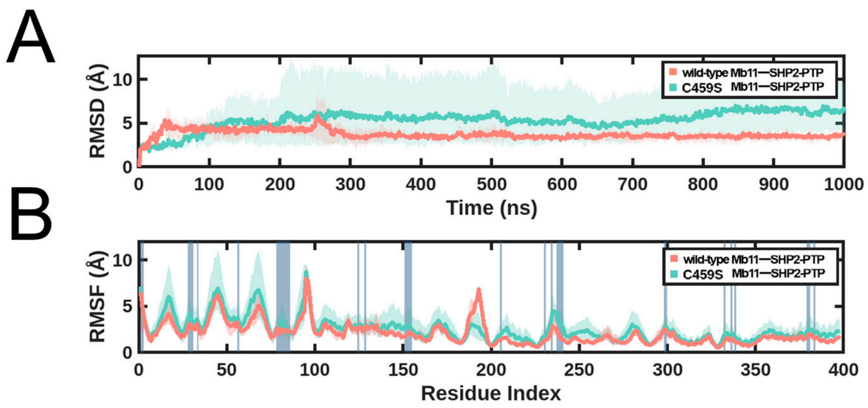

Figure S2. Comparative analysis of structural stability in wild-type and mutant Mb11–SHP2-PTP complexes. (A) RMSDs of Cα atoms for wild-type (red) and C459S mutant (green) Mb11–SHP2-PTP complexes. (B) RMSFs of Cα atoms for wild-type (red) and C459S mutant (green) Mb11–SHP2-PTP complexes, with interface residues highlighted using a semi-transparent blue background to emphasize their differential flexibility.

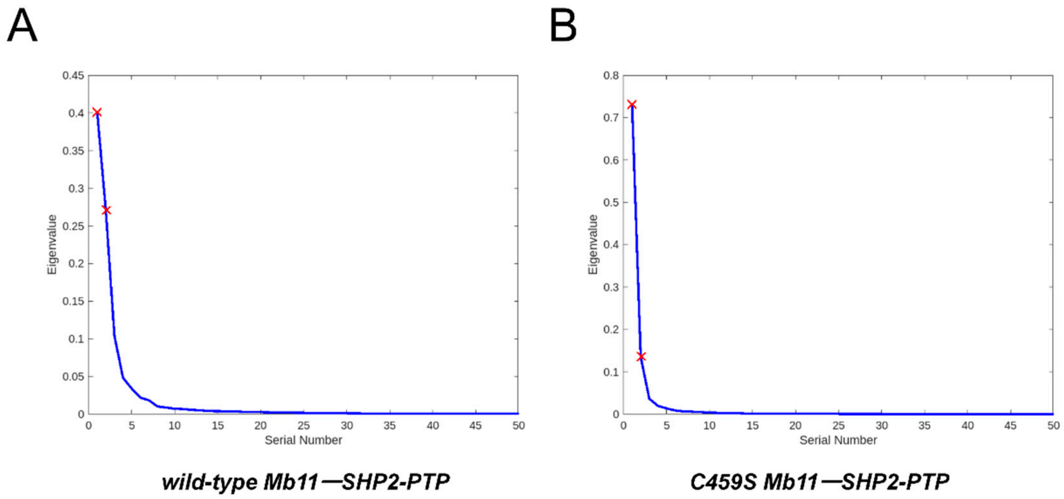

Figure S3. PCA eigenvalue distributions for the wild-type (A) and C459S mutant (B) Mb11–SHP2-PTP systems, showing the eigenvalues for PC1 to PC50. PC1 and PC2 are highlighted with red crosses.

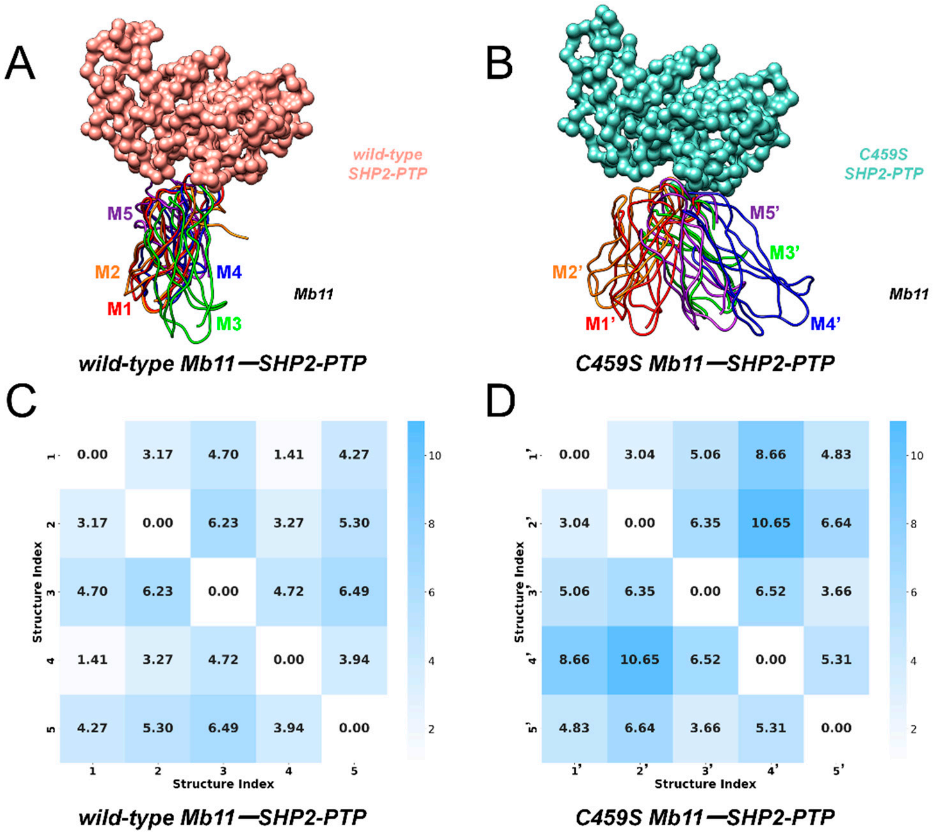

Figure S4. Representative conformations of the wild-type (A) and C459S mutant (B) Mb11–SHP2-PTP complexes, coded to match Figure 4 and highlighted in corresponding colors. The SHP2-PTP domain and its mutant are represented as red and green surfaces, respectively. RMSD heatmaps of the representative conformations for the wild-type (C) and C459S mutant (D) complexes. The color bar on the right side indicates the RMSD values.

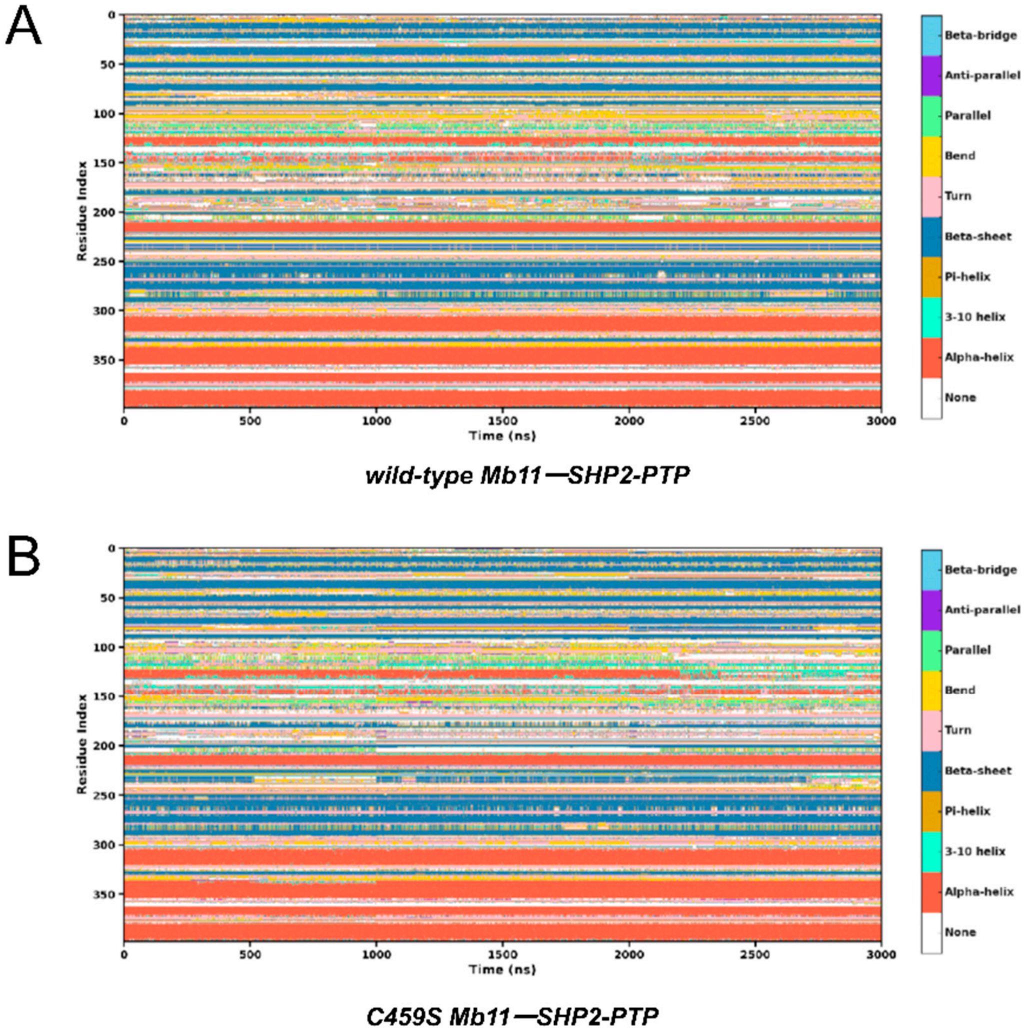

Figure S5. DSSP analysis for residues in wild-type (A) and C459S mutant (B) Mb11–SHP2-PTP. The color-coded representation of secondary structures is detailed in the legend on the right.

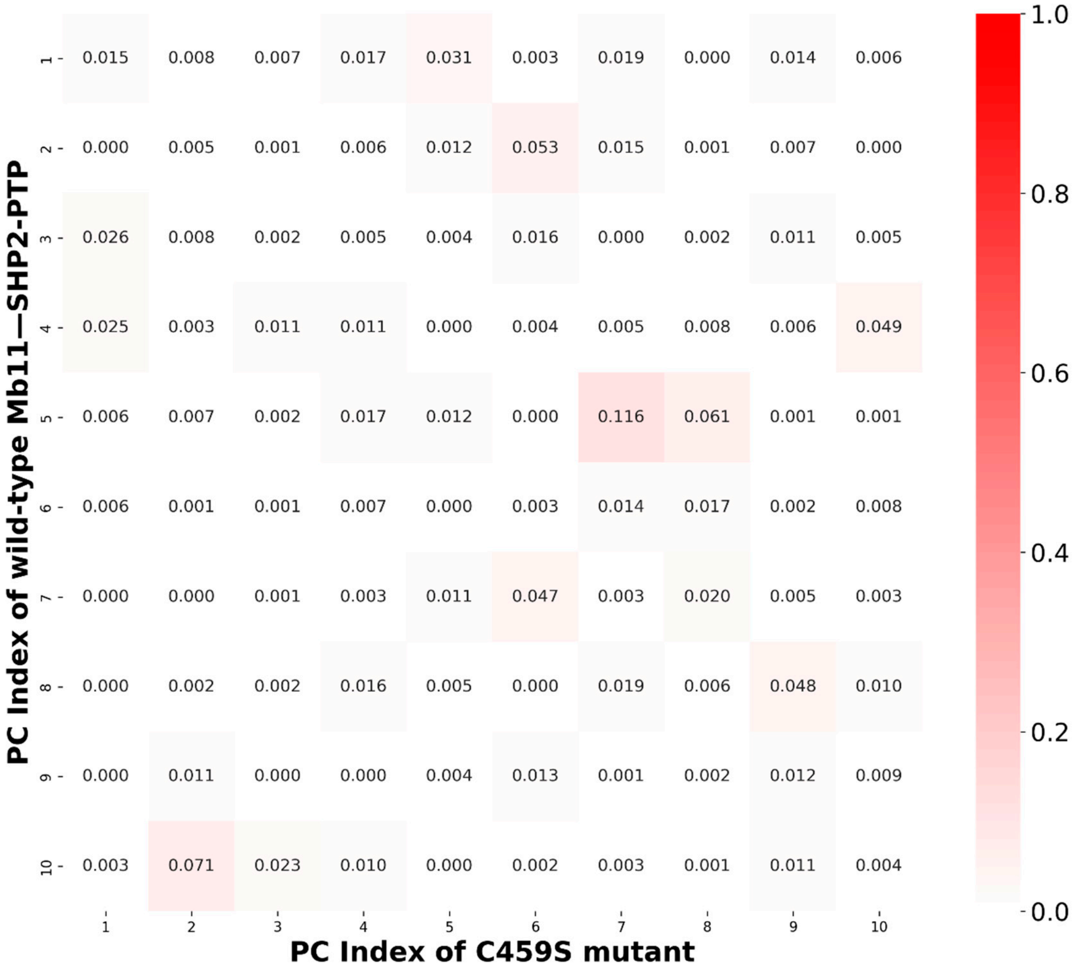

Figure S6. Heatmap of the Overlap Matrix Between the First 10 PCs of the wild-type and C459S mutant complexes. The x-axis represents the mode numbers of the C459S mutant system, while the y-axis represents the mode numbers of the wild-type system. Each cell in the matrix denotes the overlap value between the corresponding pair of modes. The color bar indicates the gradient of the overlap values, transitioning from white to red as the overlap value increases.

Table S1. Binding free energies (kcal/mol) of Mode 1 and its corresponding C459S mutant, calculated using the MM-GBSA method.

Energy component (kcal/mol)	Wild-type Mb11–SHP2-PTP	C459S Mb11–SHP2-PTP
ΔG_{gas}	-23.66 ± 46.96	-508.29 ± 88.46
ΔG_{solv}	0.81 ± 47.07	456.28 ± 77.58
$\Delta G_{binding}$	-22.85 ± 5.11	-52.00 ± 15.41

Table S2. Binding free energies (kcal/mol) of Mode 2 and its corresponding C459S mutant, calculated using the MM-GBSA method.

Energy component (kcal/mol)	Wild-type Mb11–SHP2-PTP	C459S Mb11–SHP2-PTP
ΔG_{gas}	-285.42 ± 57.27	-364.08 ± 48.95
ΔG_{solv}	251.52 ± 53.00	287.99 ± 47.38
$\Delta G_{binding}$	-33.91 ± 6.55	-76.09 ± 4.84

Table S3. Binding free energies (kcal/mol) of Mode 3 and its corresponding C459S mutant, calculated using the MM-GBSA method.

Energy component (kcal/mol)	Wild-type Mb11–SHP2-PTP	C459S Mb11–SHP2-PTP
ΔG_{gas}	-482.92 ± 67.09	-498.85 ± 95.77
ΔG_{solv}	434.49 ± 59.34	461.74 ± 90.15
$\Delta G_{binding}$	-48.43 ± 11.97	-37.11 ± 10.62

Table S4. Detailed residue composition of Loops M1, M2, P1, and P2.

Loop	Residue Constituents
M1	D25-Y35
M2	W78-E82
P1	N150-L156
P2	T229-V241
